# Supplementary material for: Impact of miR-1/miR-133 Clustered miRNAs: PFN2 Facilitates Malignant Phenotypes in Head and Neck Squamous Cell Carcinoma
Source: Biomedicines. 2022 Mar 12;10(3):663. doi: 10.3390/biomedicines10030663 (PMC8944972; doi:10.3390/biomedicines10030663)
Supplement: Supplementary file 1 [file biomedicines-10-00663-s001.zip › 20220216 supplementary figures miR-133ab_miR-1-3p_2-6_PFN2.pdf]

Figure S1

SAS

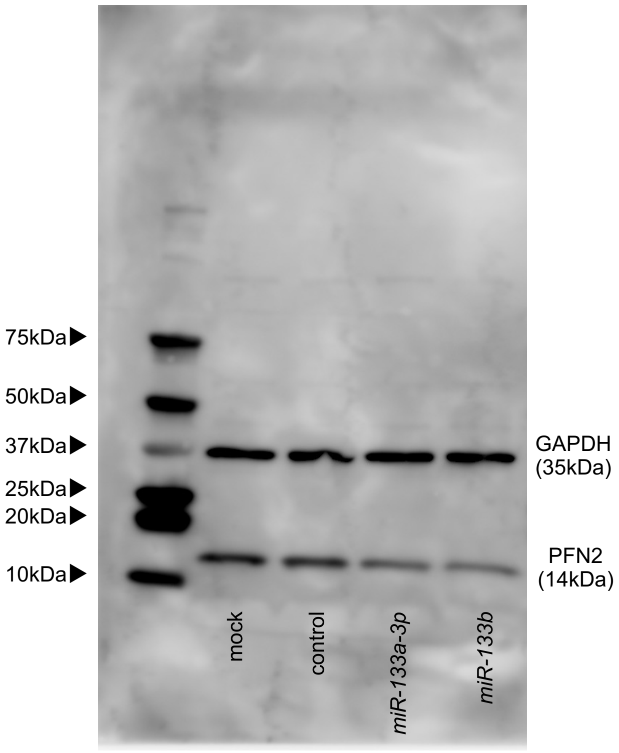

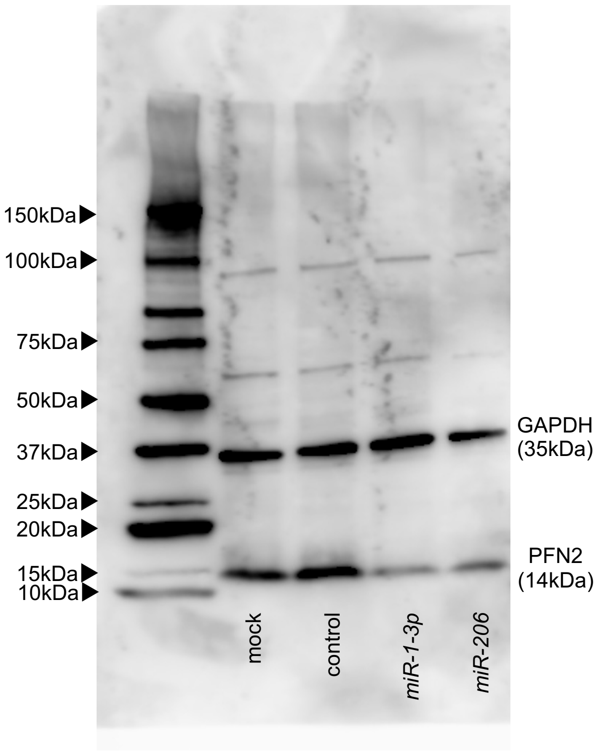

Sa3

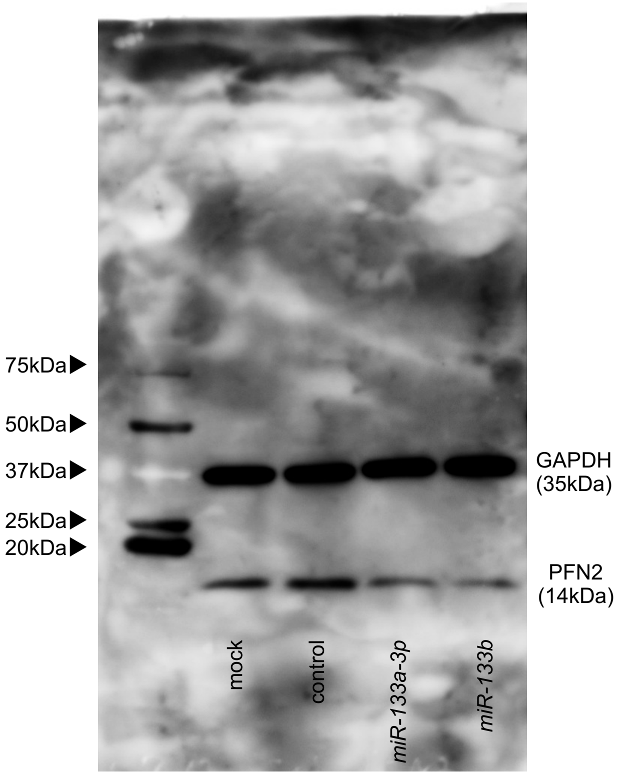

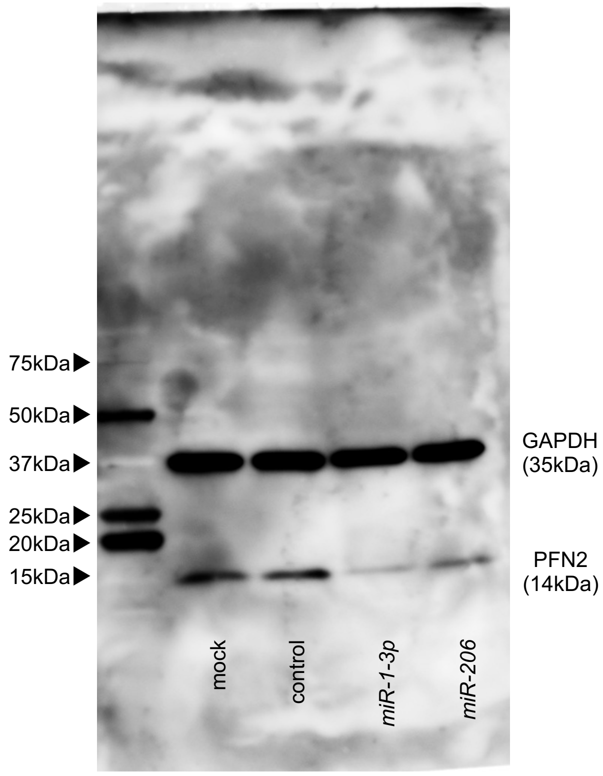

Figure S1. Full blots of Figure 6A. Expression of PFN2 after *miR-133a-3p/miR-133b* and *miR-1-3p/miR-206* transfection HNSCC cells.

Figure S2

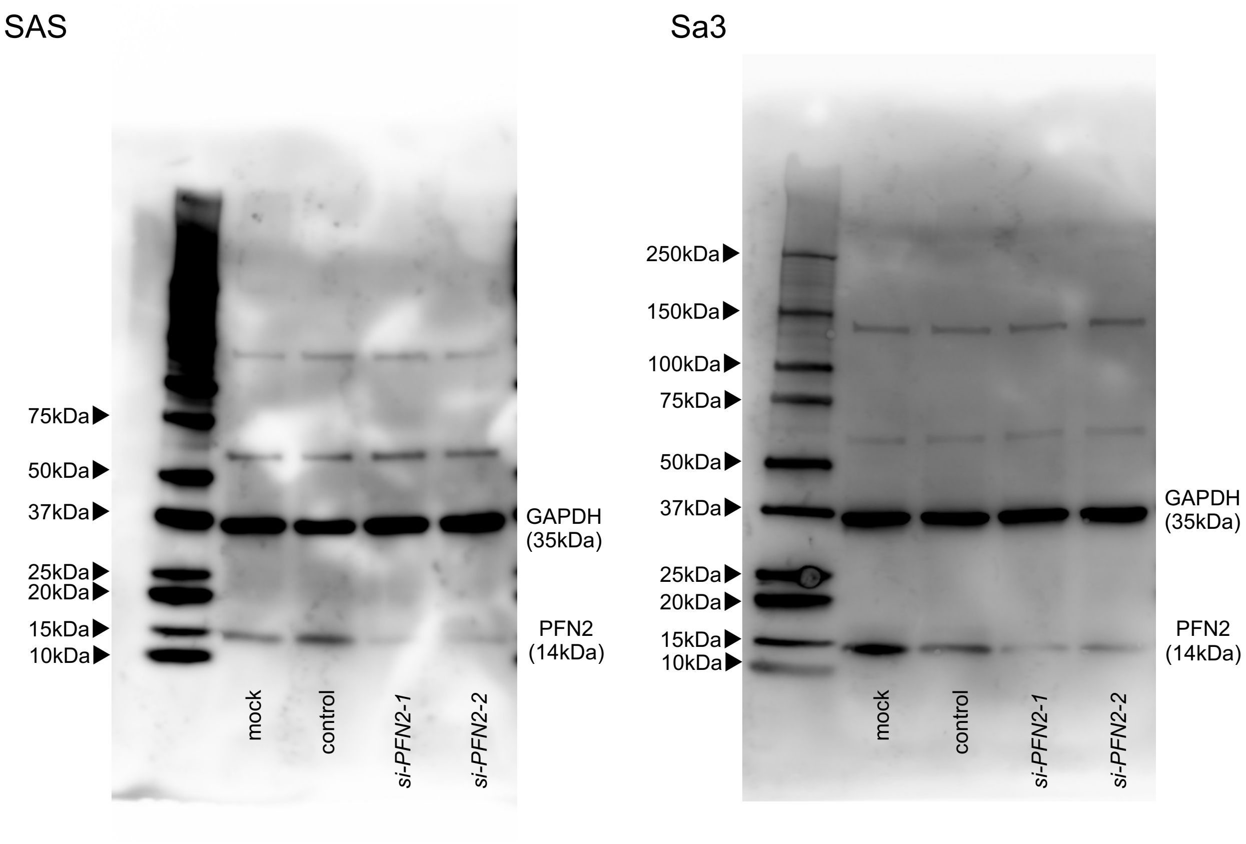

Figure S2. Full blots of Figure 8B. Expression of PFN2 after *siPFN2* transfection HNSCC cells.

Figure S3

migration assay

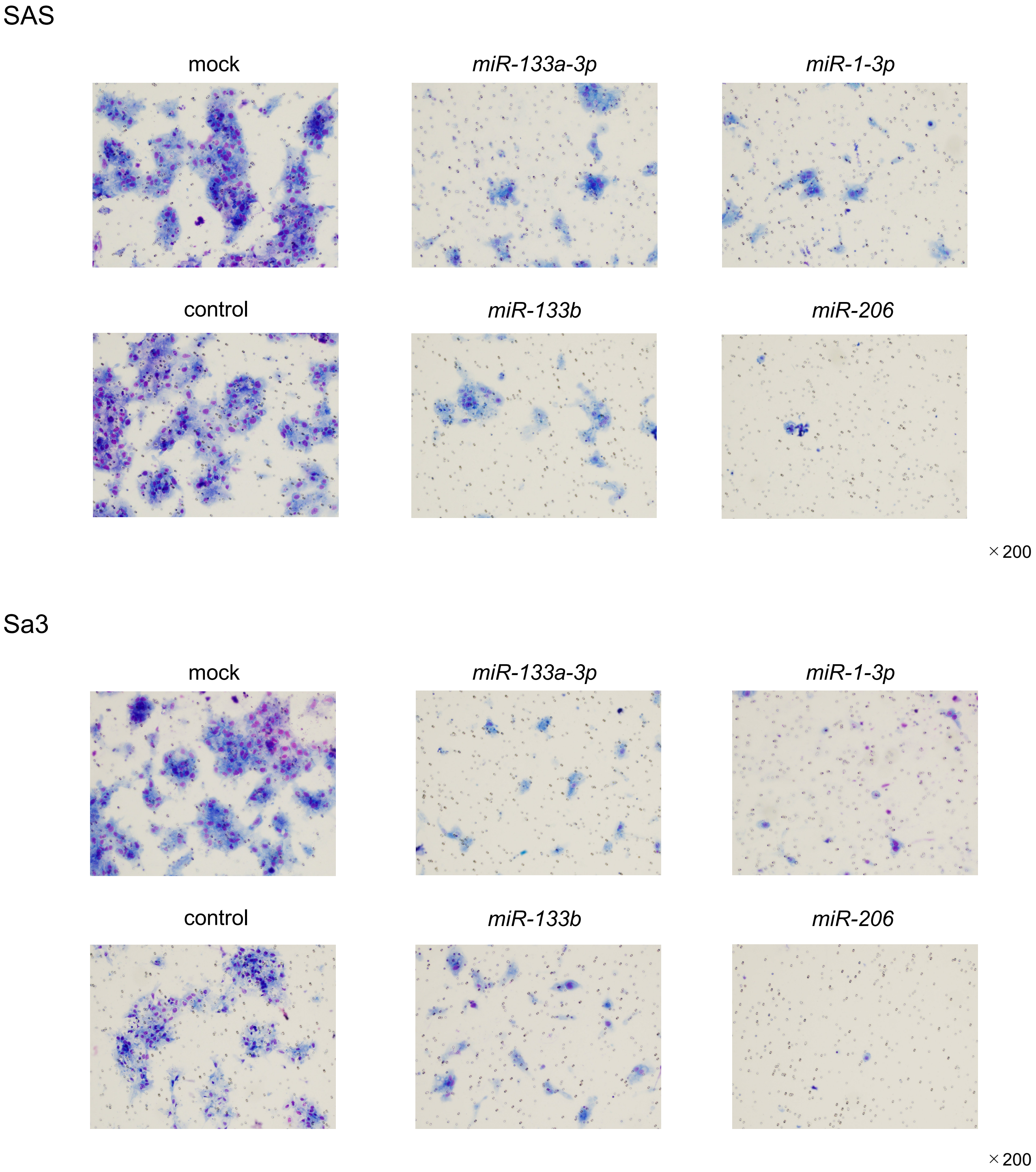

Figure S3. Photomicrographs of cells in migration assays.
Typical images of cells in migration assays following *miR-133a-3p/miR-133b* and *miR-1-3p/miR-206* transfection in HNSCC cells.

Figure S4

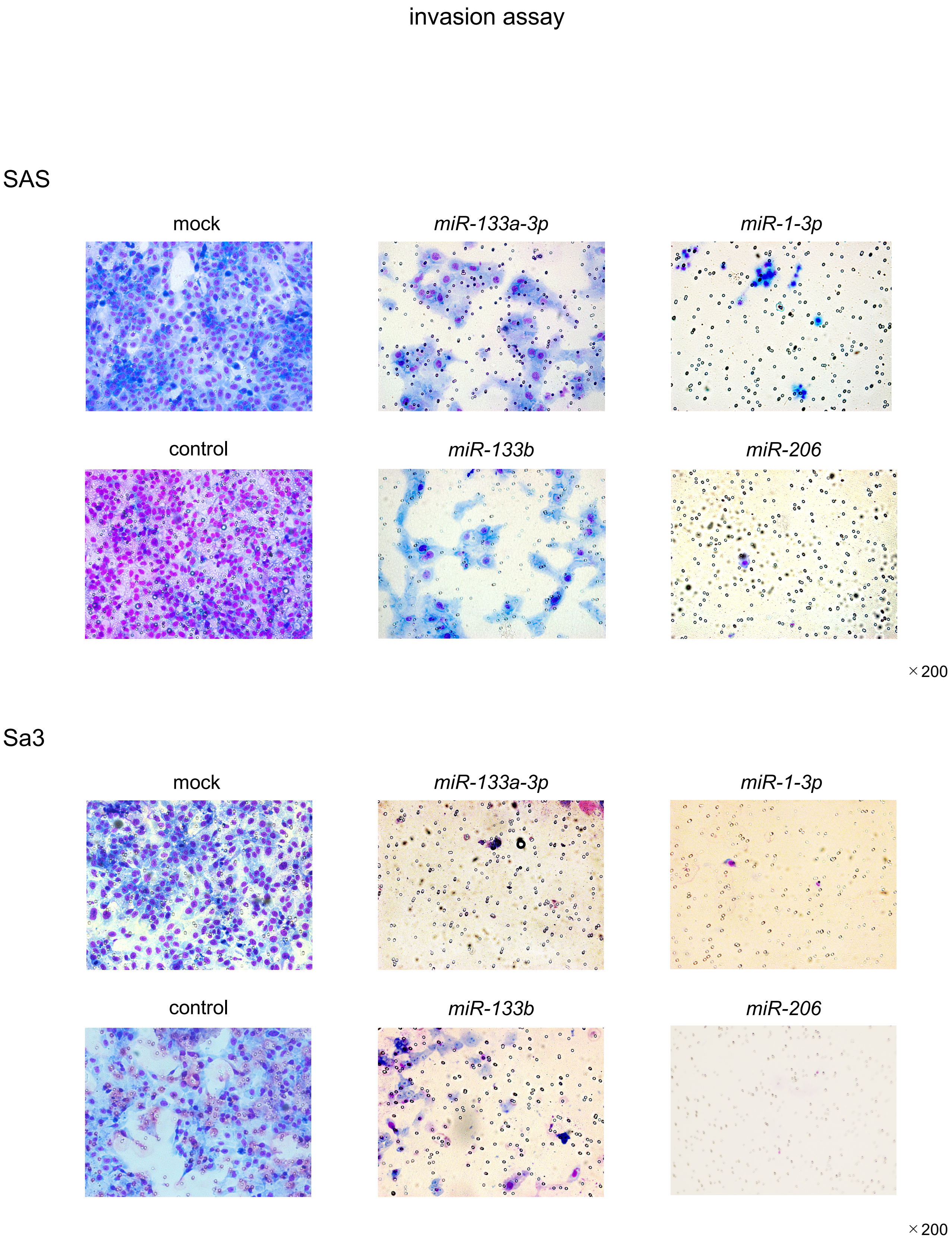

Figure S4. Photomicrographs of cells iin invasion assays.
Typical images of cells in invasion assays following *miR-133a-3p/miR-133b* and *miR-1-3p/miR-206* transfection in HNSCC cells.

Figure S5

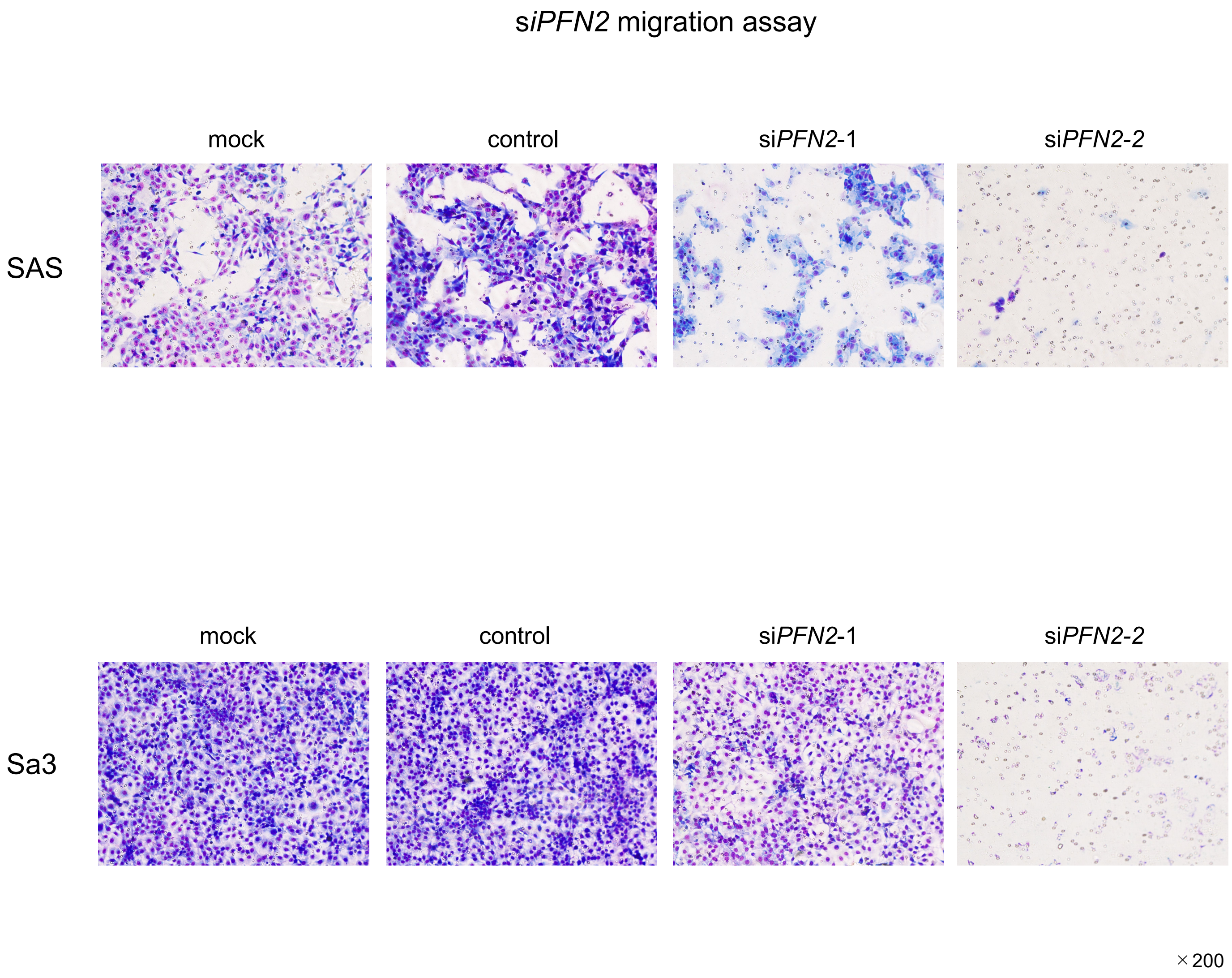

Figure S5. Photomicrographs of cells in migration assays.
Typical images of cells in migration assays following *siPFN2* transfection in HNSCC cells.

Figure S6

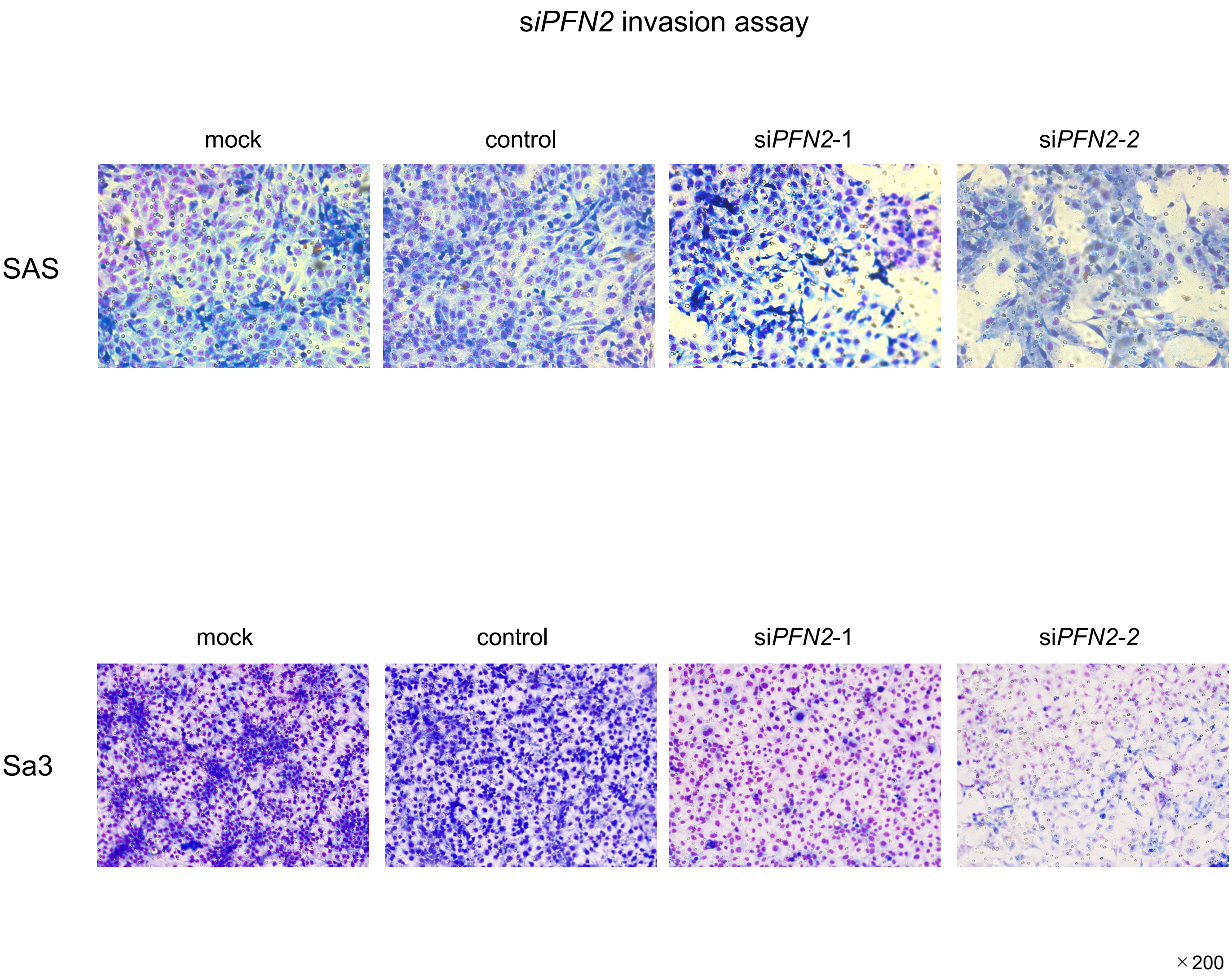

Figure S6. Photomicrographs of cells in invasion assays.
Typical images of cells in invasion assays following *siPFN2* transfection in HNSCC cells.
